# Increasing COVID-19 Vaccination Coverage for Newcomer Communities: The Importance of Disaggregation by Language

**DOI:** 10.4269/ajtmh.22-0724

**Published:** 2023-04-17

**Authors:** Abigail Steiner, Kristine Knuti Rodrigues, Nadège Mudenge, Janine Young, Rasulo Rasulo, Colleen Payton, Malini DeSilva, Jeremy Michel, Mary Fabio, Katherine Yun

**Affiliations:** ^1^ Denver Health & Hospital Authority, Denver, Colorado;; ^2^ Children’s Hospital of Philadelphia, Philadelphia, Pennsylvania;; ^3^ UC San Diego School of Medicine, San Diego, California;; ^4^ Thomas Jefferson University, Philadelphia, Pennsylvania;; ^5^ Moravian University, Bethlehem, Pennsylvania;; ^6^ HealthPartners Institute, Bloomington, Minnesota

## Abstract

The COVID-19 pandemic has disproportionately affected refugee, immigrant, and migrant populations. Vaccines are essential for decreasing transmission and severity of COVID-19 infection. Understanding differences in vaccination coverage based on preferred language is crucial for focusing efforts to decrease COVID-19–related disparities. Four sites in the Minnesota Center of Excellence in Newcomer Health collaboratively evaluated completion of primary COVID-19 vaccination series on or before December 31, 2021, for patients who were 12 years or older on June 30, 2021, by preferred language. The non-English/non-Spanish speaking population included 46,714 patients who spoke 174 languages; COVID-19 vaccination coverage by language ranged from 26.2% to 88.0%. Stratifying vaccination coverage by specific language is a critical first step toward dismantling disparities and shaping interventions that best meet the needs of communities served.

## INTRODUCTION

Immigrant communities in the United States have historically experienced multiple barriers to care, leading to persistent health disparities.^1^^,^^2^ Previous studies have documented delayed time to care, decreased access to healthcare services, and lower rates of health insurance coverage for immigrant patients who speak languages other than English or Spanish.^2^^,^^3^ Early in the COVID-19 pandemic, higher-than-typical risk of infection and hospitalization among several immigrant and other communities speaking non-English, non-Spanish (NENS) languages was observed.^4^^–^^7^ Risk was attributed to lack of access to testing and personal protective equipment, especially for the in-person essential workforce. Further, several immigrant communities lacked access to information in preferred languages and were excluded from pandemic relief programs.^8^^–^^11^

After COVID-19 vaccines received emergency use authorization in the United States, concerns were raised about equity in vaccination access for immigrant communities.^12^^,^^13^ Major barriers included English-only electronic vaccination scheduling portals and mass vaccination sites with hours and locations inaccessible to many in-person essential workers. Initial steps to address these disparities often prioritized Spanish-speaking communities. Although profoundly important, English/Spanish-only approaches nonetheless exclude the ∼26.5 million US residents who speak other languages.^14^ Additionally, many efforts to understand differences in COVID-19 vaccination coverage and shape subsequent interventions have focused on race and ethnicity; these social constructs are used differently in the United States relative to many other countries.^15^^,^^16^ Racial categories fail to account for differences in identity, culture, and experience of individuals born elsewhere.^17^

The aim of this project was to examine COVID-19 primary series vaccination coverage during the first year of vaccine availability by language group to inform future interventions for NENS primary care patients within four large health systems in the United States.

## MATERIALS AND METHODS

A cross-sectional analysis of COVID-19 vaccination status among patients ≥ 12 years as of June 30, 2021, was performed using vaccination data from four health systems participating in Minnesota Department of Health’s Center of Excellence in Newcomer Health, sponsored by the CDC. The four sites included an integrated safety net health system in Denver, CO; a children’s hospital in Philadelphia, PA; a healthcare system serving Minnesota and Western Wisconsin; and a university health system in Philadelphia, PA. The study population was limited to patients ≥ 12 years of age as of June 30, 2021, to ensure adequate time to receive the primary series (vaccination for 5- to 11-year-old children was authorized on October 29, 2021).^18^ Each participating site received expedited, exempt, or not Human Subjects Research status for this analysis from their local institutional review board. Vaccination records were extracted from each site’s electronic health record (EHR)—all sites use Epic®—for patients seen in primary care (defined as Internal Medicine, Family Medicine, Pediatrics, or Obstetrics and Gynecology) from January 1, 2019, to December 31, 2021. Vaccination records at each site are linked to respective local and state vaccination registries via automated connections managed by the EHR. This allowed for default inclusion of vaccinations administered within and outside of each healthcare system. The primary outcome in this analysis was completion of the primary COVID-19 vaccination series prior to December 31, 2021. Completion of the primary series was defined as two doses of an mRNA vaccine, two doses of FDA/WHO approved recombinant vaccines, or a single dose of the Janssen recombinant vaccine.

The primary independent variable was preferred language documented in the EHR as indicated by patients at intake into care. Patients without a preferred language in the EHR and patients with a language listed that was not documented as a known language were excluded from the analysis. Patients whose preferred language was English were compared with patients whose preferred language was Spanish or a language other than English or Spanish. Languages were then disaggregated, and vaccination status was examined for the 25 largest language groups identified across all sites. Vaccination status was also analyzed by both age group and preferred language at one site to assess whether there were differences between language groups for patients aged 65+ years because older patients are at higher risk of worse outcomes following COVID-19 infection.^19^

Descriptive statistics using frequencies and proportions were used to describe the differences in COVID-19 vaccination status by language. Risk ratios and associated 95% CIs were calculated to understand the likelihood of having completed a primary COVID-19 vaccination series by NENS language group compared with the English- and Spanish-speaking patient group. Data were analyzed using SAS Enterprise Guide 7.1.

## RESULTS

During the study period, 1,345,848 patients with 176 preferred languages were seen for primary care across the four participating health systems. Of those, 64.9% completed their primary COVID-19 vaccination series by December 31, 2021 (Table 1). This was similar for subpopulations whose preferred language was English (64.9%), Spanish (65.7%), and NENS (63.8%).

**Table 1:** COVID-19 primary series vaccination status among current primary care patients ages ≥ 12 years across four sites in 2020–2021: English, Spanish, and non-English/non-Spanish language subgroups

Groups	Total population	Vaccinated, %
Overall*	1,345,848	64.91
Overall non-English	103,581	64.85
Overall non-English/non-Spanish†	46,714	63.80
English	1,242,267	64.92
Spanish	56,867	65.71

Sites include Denver Health and Hospital Authority (Denver, CO), Children’s Hospital of Philadelphia (Philadelphia, PA), Thomas Jefferson University (Philadelphia, PA), and HealthPartners (Minneapolis, MN).

* Inclusion criteria for this study was anyone aged 12 years or over on or before June 30, 2021, seen for primary care (internal medicine, family medicine, pediatrics, OBGYN) between January 1, 2019 and December 31, 2021. Records were excluded if preferred language was unknown, missing, or declined.

† A total of 174 non-English, non-Spanish languages were identified in the study cohort.

There were 41,894 NENS-speaking patients who identified one of the 25 most common languages across all four sites as their preferred language (89.7% of NENS population). Completion of a primary COVID-19 vaccination series ranged from 42.7% to 88.0% by language group (Table 2). Compared with the English- and Spanish-speaking population, the NENS population speaking the 25 most common languages were 2% less likely to have completed a primary COVID-19 vaccination series. This ranged from 34% less likely to 35% more likely across these NENS language groups.

**Table 2:** Completion of COVID-19 vaccine primary series by December 31, 2021, among current primary care patients aged ≥ 12 years whose preferred language is among the most common 25 non-English/non-Spanish languages across four sites, 2020–2021*†

Language	*N*	Vaccinated, %	Risk ratio (95% CI)‡
Overall	41,894	63.87	0.98 (0.976–0.99)
Amharic	2,579	69.02	1.06 (1.04–1.09)
Arabic§	2,523	57.27	0.88 (0.85–0.91)
Bengali	236	75.85	1.17 (1.09–1.26)
Burmese	552	66.85	1.03 (0.97–1.09)
Cambodian	1,673	83.38	1.28 (1.26–1.31)
Cantonese	718	83.98	1.29 (1.25–1.34)
Chineseǁ	216	67.13	1.03 (0.94–1.14)
Dari¶	264	77.65	1.20 (1.12–1.28)
Farsi#	341	75.07	1.16 (1.09–1.23)
French	1,077	61.00	0.94 (0.90–0.99)
Hindi	293	70.65	1.09 (1.01–1.17)
Hmong	2,202	68.98	1.06 (1.03–1.09)
Karen	1,384	61.42	0.95 (0.91–0.99)
Korean	464	81.68	1.26 (1.21–1.31)
Lao	849	81.39	1.25 (1.21–1.29)
Mandarin	1,489	75.35	1.16 (1.13–1.19)
Nepali	1,515	77.16	1.19 (1.16–1.22)
Oromo	1,862	63.16	0.97 (0.94–1.00)
Portuguese	250	63.20	0.97 (0.89–1.07)
Russian	1,805	42.66	0.66 (0.62–0.69)
Somali	12,094	49.67	0.76 (0.75–0.78)
Swahili**	653	44.41	0.68 (0.63–0.75)
Tibetan	225	88.00	1.35 (1.29–1.42)
Tigrinya	696	65.80	1.01 (0.96–1.07)
Vietnamese	5,934	79.69	1.23 (1.21–1.24)

* These 25 languages were spoken by 89.7% of non-English, non-Spanish speaking individuals.

† High rates of vaccination coverage in some language groups may reflect the circumstances of communities with a large proportion of newcomers for whom COVID-19 vaccination was strongly recommended or required for US entry or adjustment of status.

‡ The ratio of the % vaccinated in the individual language group compared with the % vaccinated in the English and Spanish speaking control group (64.95%).

§ Moroccan Arabic, Sudanese Arabic, and Arabic (not otherwise specified) were all categorized as Arabic.

ǁ Chinese was not combined with Mandarin or Cantonese due to lack of specificity.

¶ Dari was not combined with Farsi because Dari speakers from Afghanistan are not always able to communicate with Farsi interpreters from Iran.

# Persian and Farsi were both categorized as Farsi.

** Type of Swahili spoken (e.g., Swahili spoken in the Democratic Republic of Congo vs. Swahili spoken in Tanzania) was not specified in the electronic health record of participating health systems.

At one site, vaccination status was analyzed by preferred language for 1,441 individuals aged 65+ years (Table 3). Among patients aged 65+ years, the percentage vaccinated ranged from 57.6% to over 95% for different language groups.

**Table 3:** Completion of COVID-19 vaccine primary series by December 31, 2021, among current primary care patients aged ≥ 12 years whose preferred language is among the most common 15 non-English/non-Spanish languages within a single health system, overall population (*N* = 7,372), and for those age ≥ 65 years (*N* = 1,441), 2020–2021

Language*	Overall population				Age ≥ 65 years	
	Mean age, years	Median age (IQR)	*N*	Vaccinated, %	*N*	Vaccinated, %
Amharic	44.5	42 (33–56)	999	68.2	164	70.1
Arabic	42.0	40 (29–56)	1,373	57.5	207	74.4
Burmese	34.5	36 (22–43)	331	58.3	9	77.8
Chinese	55.2	63 (32–78)	139	69.1	66	78.8
Dari	33.3	31 (23–37)	214	82.2	17	> 95†
Farsi	45.4	42 (31–62)	175	75.4	37	91.9
French	41.0	38 (29–53)	412	60.4	60	71.7
Mandarin	61.7	71 (44–80)	182	81.3	103	89.3
Nepali	42.4	39 (29–56)	645	73.3	94	78.7
Oromo	37.6	36 (28–46)	190	66.3	13	69.2
Russian	63.3	68 (51–81)	641	47.7	370	57.6
Somali	36.1	35 (21–47)	525	53.3	36	63.9
Swahili	32.7	30 (20–42)	257	40.9	12	83.3
Tigrinya	41.6	38 (26–57)	370	61.4	64	78.1
Vietnamese	45.6	48 (28–62)	919	77.8	189	90.5

IQR = interquartile range.

* To prevent deductive identification of vaccination status for members of small communities, only the 15 largest language subgroups are shown. These languages were spoken by 78.1% of non-English, non-Spanish–speaking individuals.

† Total vaccinated was more than 95% of the subpopulation.

## DISCUSSION

In this evaluation of 46,714 patients whose preferred language is NENS, there was a wide variation in the percentage of the population with a completed COVID-19 primary vaccination series by language. This project demonstrates that grouping patients who speak languages other than English or Spanish as one entity instead of stratifying by specific languages masks disparities. Furthermore, wide variation in vaccination coverage by language group was observed even when focusing only on patients aged 65+ years who—along with immunocompromised individuals—are a priority population for COVID-19 vaccination interventions.^19^

In lieu of examining language data, health equity assessments often focus predominantly on race and/or ethnicity. Although important, these strategies do not allow for allocation of language-related resources and can also obscure between-group differences for NENS communities. As other health equity teams have noted, the federal categories for race and ethnicity are insufficient for self-identification of many newcomer communities, including but not limited to Asian and Pacific Islander subgroups, Hispanic/Latino subgroups, and individuals from the Middle East and North Africa.^17^ Additionally, many individuals with a NENS preferred language do not identify with US racial groupings. Race is therefore incomplete for analyzing the impact of vaccination programs for many NENS patients.

For this reason, health systems interested in promoting vaccination equity should ask patients their preferred language, document preferred language in the EHR, and routinely examine vaccination status by specific language subgroups.^20^ Accurate documentation of language facilitates health systems’ ability to identify communities that should be prioritized for outreach, education, and access in preferred languages. Across the four sites that participated in this analysis, examination of baseline data for specific language groups allowed newcomer health teams at participating sites to advocate for a wide range of multilingual vaccination outreach, education, and other access initiatives. Site-specific strategies included recruitment of bilingual staff and volunteers, adjustment of the geographic distribution of mobile vaccination clinics, creation of multilingual scheduling protocols for languages other than English, partnerships with ethnic community–based organizations, and cultivation of relationships with selected trusted messengers, such as religious leaders.

This analysis was subject to several limitations. Vaccination records may be incomplete if patients were vaccinated at sites with lags in reporting to state vaccination registries or were vaccinated in other states. However, data were pulled more than 1 month after the end of the study period to help account for data lag issues. Potential misclassification of preferred language is another potential limitation because it is dependent on how preferred language is recorded when patients present for care. For example, if a pediatric patient who speaks English is accompanied by a relative who speaks Mandarin, their preferred language may be entered as “English” in the EHR. However, their caregiver requires vaccine education and outreach in Mandarin. This analysis also uncovered problems with language lists within the EHR: lists at some sites need greater specificity (e.g., “Chinese” may indicate Mandarin or other varieties of Chinese, such as Cantonese), lists often need to be more comprehensive (e.g., one site allowed only 100 options and re-coded all other languages as “other”), and lists often need to be tailored to regional differences in immigrant communities represented (e.g., specific languages spoken by indigenous groups may need to be added). Data presented here do not consider time of US arrival of specific subpopulations and legal status type, so some subpopulations may have had more robust uptake of COVID vaccinations due to US immigration requirements. It is also important to recognize that there may be differences in vaccination by language in different parts of the country. For this analysis, all patients were grouped by language irrespective of where they live. Future analyses could work to understand variation within language groups across sites. Finally, this analysis was limited to addressing language groups; however, efforts to support the health of newcomer communities may also benefit from knowing patients’ countries of origin. For example, preferred media and trusted messengers for Swahili-speaking populations who are Tanzanian may be very different from those for Swahili-speaking populations who have origins in the Democratic Republic of Congo.

These results have clinical, structural, and policy implications. Lower rates of vaccination in subpopulations speaking particular NENS languages could result in relative increased illness burden in groups already disadvantaged by barriers to accessing health systems supports and information in primary languages. Effective efforts to decrease health disparities and improve access to healthcare require adequate identification of these disparities. Health systems and EHR vendors should ensure that preferred language fields within the EHR are comprehensive and unambiguous. They should also ensure staff training includes guidance on standard scripted questions and accurate documentation of preferred language. Data systems should be programmed to examine individual language groups rather than English, Spanish, and “other.” Analyzing vaccination coverage by preferred language can significantly inform public health efforts by illuminating disparities in vaccination coverage and can support the tailoring of interventions to increase vaccination in specific subpopulations with lower vaccination rates.
